# Evaluation of the reliability of the upper lip bite test and the modified mallampati test in predicting difficult intubation under direct laryngoscopy in apparently normal patients: a prospective observational clinical study

**DOI:** 10.1186/s12871-022-01855-7

**Published:** 2022-10-10

**Authors:** Lin-yu Wang, Kang-da Zhang, Zhi-hua Zhang, Dan-xu Zhang, Huan-liang Wang, Feng Qi

**Affiliations:** 1 Department of Anesthesiology, Qilu Hospital of Shandong University, 107 Wenhua West Road, Lixia District, Jinan City, Shandong Province China; 2 Shenzhen Research Institute of Shandong University, A301 Virtual University Park in South District of Shenzhen, Shenzhen, China; 3 Department of Anesthesiology, Second Affiliated Hospital of Hainan Medical University, Haikou, Hainan China

**Keywords:** Upper lip bite test, Modified mallampati test, Difficult intubation, Direct laryngoscopy

## Abstract

**Background and aims:**

Difficult endotracheal intubation is one of the most challenging operations in anesthesia. How to better predict difficult airway and make corresponding preparations to reduce the occurrence of accidents is a difficult task faced by anesthesiologists every day. This study decide to evaluate the value of the Upper Lip Bite Test (ULBT) and the Modified Mallampati Test (MMT) in predicting difficult intubation under direct laryngoscopy and find out the most intuitive and simple method to predict difficult intubation under direct laryngoscopy in apparently normal patients.

**Patients and methods:**

This descriptive-analytical study was performed on 450 patients for elective surgery under general anesthesia requiring endotracheal intubation. The ULBT and MMT grading were evaluated preoperatively and Cormack and Lehane’s (CL) classification was recorded on the day of surgery during intubation under direct laryngoscopy. The accuracy, sensitivity, specificity, positive predictive value (PPV), negative predictive value (NPV), likelihood ratio (LR), Youden index and area under ROC curve of ULBT and MMT respectively and in combination were calculated and compared. And the consistency between the total scores of ULBT and MMT combined in different ways and CL grading was counted.

**Results:**

Of the 450 patients, 69 (15.3%) were classified as difficult cases of direct laryngoscopy. The accuracy, sensitivity, specificity, PPV and NPV of ULBT were 81.33, 11.59, 93.96, 25.81, 85.44%; and those the corresponding values for MMT were 66.22, 62.32, 69.29, 26.88 and 91.03%. A combination of ULBT and MMT did not improve the sensitivity in the sample tested. The combined total scores of ULBT and MMT in both ways were less consistent with CL grading in predicting difficult intubation under direct laryngoscopy.

**Conclusion:**

Based on findings of current study, we conclude that ULBT and MMT for difficult intubation have only poor to moderate discriminative power when used alone.

The combination of the two tests in fractional form is also not a good predictor of difficult intubation under direct laryngoscopy.

**Trial registration:**

Chinese Clinical Trial Registry, ChiCTR2100052987, Registered 07 November 2021, http://www.chictr.org.cn

**Supplementary Information:**

The online version contains supplementary material available at 10.1186/s12871-022-01855-7.

## Introduction

In recent years, with the updating of anesthesia equipment, the level of anesthesia technology has been improved. The clinical application of general anesthesia and endotracheal intubation is becoming more and more widespread, but the incidence of difficult intubation as high as 1–18% is still a difficult situation for clinical anesthesiologists [1]. Failure of oxygenation or an unexpectedly difficult airway can result in brain hypoxia, brain damage and even death [2].

By being able to anticipate the presence of a difficult airway, we will be able to plan for appropriate equipment, experienced personnel, and alternative airway management strategies, such as endotracheal intubation with spontaneous breathing and awareness [3].

Therefore, it is important to have a simple and direct prediction of difficult airways in seemingly normal patients, but there is no standard test to assess and predict. Many researchers have attempted to predict difficult intubation by using simple bedside physical examinations, such as the upper lip bite test (ULBT) and the modified mallampati test (MMT) [4]. It has been reported that ULBT appears to be a useful bedside test for predicting difficult airways with moderate sensitivity and high specificity, with higher accuracy than MMT [5–7]. Any test needs to be proven over and over again. The purpose of this study was to evaluate the value of these two tests in predicting difficult intubation under direct laryngoscopy and find out the most intuitive and simple method to predict difficult intubation under direct laryngoscopy in patients without risk factors for difficult airways.

## Materials and methods

### Study design

This was a prospective observational, single-centre study which was conducted in Qilu Hospital of Shandong University between 2021 and 2022. The study was approved by the Medical Ethics Committee of Qilu Hospital of Shandong University (Approval Document No. 2020 (095)) and registered in The Chinese Clinical Trial Registry (Registration Number: ChiCTR2100052987) on 07/11/2021. All participating patients were informed of the purpose and process of the study and signed an informed consent for anesthesia.

### Participants

Four hundred and fifty adults aged between 18 to 75 years old with American Society of Anesthesiologists (ASA) physical status I-II, who were scheduled to undergo endotracheal intubation general anesthesia for orthopedics, thoracic, neurosurgery, and general surgery were enrolled in this prospective observational study. Patients who were unwilling to participate, patients with body mass index (BMI) > 35 kg/m^2^, muscle weakness, limited mouth opening, and a large tongue, patients without teeth or with dentures, patients with limited neck mobility and mandible, emergency surgery patients, and subjects who could not cooperate were excluded from the study.

### Definitions

Difficult intubation: Endotracheal intubation by an experienced anesthesiologist requires more than three attempts, regardless of the presence or absence of airway pathology.

MMT: Patient was asked to sit up with his mouth open as much as possible and to stick out his tongue without making a sound. Oropharyngeal structures are visualized and classified with the help of a flashlight [8–10].

Class I: soft palate, fauces, uvua and pillars

Class II: soft palate, fauces and uvula

Class III: Soft palate and base of uvula

Class IV: Hard palate only

I & II are considered as predictors of easy intubation.

III & IV are considered as predictors of difficult intubation.

ULBT: ULBT evaluates the range and freedom of mandibular movement and the architecture of the teeth [11]. In this examination, patients were asked to bite their upper lip with lower incisors and were graded accordingly by the upper lip mucosa as the boundary [9].

Class I: Lower incisor can bite the upper lip above the vermillion line

Class II: Lower incisor can bite the upper lip below the vermillion line

Class III: Lower incisors can not bite upper lip

Class I &II are predictive of easy intubation whereas Class III suggests difficult intubation [12].

CL classification: After adequate muscle relaxation, the patient is placed in the sniffing position, but no external laryngeal pressure is applied [9]. On direct laryngoscopy glottis view was classified according to CL classification.

Class I: full view of glottis is seen

Class II: Glottis partly exposed, only posterior commissure is seen

Class III: Only epiglottis is seen

Class IV: Epiglottis is not seen

Class I and II are considered as easy intubation and III and IV as difficult Intubation [8].

### Anesthesia management

The day before surgery, an anesthesiologist visited the enrolled patients and recorded all data relevant to the subjects, including type of surgery, age, gender, weight, height, BMI, ASA, medical history and the grade of ULBT and MMT on prepared forms. Subsequently, the second anesthesiologist with more than 3 years experience in anesthesia performed a direct laryngoscopy on the day of surgery after sufficient muscle relaxation induced by anesthesia and determined each subject’s CL grading [13].

### Endpoints

The combination of ULBT and MMT scores for predicting CL grading under direct laryngoscopy was the main endpoint of the study. Investigating the effectiveness of ULBT and MMT in the prediction of difficult airways was the secondary endpoint of the study.

### Statistical analysis

SPSS 25 was used to analyze the data. Quantitative results, such as age, weight and height, BMI are presented in the form of mean and standard deviation. Frequency and percentage of qualitative variables such as gender and ASA status were calculated. Data for each continuous variable were analyzed for normal distribution using the Kolmogorov-Smirnov test combined with histograms and P-P plots. Analysis of continuous variable with a normal distribution was performed using the two-tail Student’s t-test and the Chi-square test was used for categorical variables. Paired Chi-square test (McNemar-Bowker Test) was used to test the correlation between the two variables in the paired design of multi-classification ordered variables. Accuracy, sensitivity, specificity, PPV and NPV were calculated for MMT and ULBT, while maintaining CL grading as the gold standard. The *p*-value and Kappa value of paired Chi-square test (McNemar-Bowker Test) for the total score of MMT and 2-fold ULBT and CL grading and for the total score of ULBT and 2-fold MMT and CL grading was counted for predicting consistency of difficult intubation. *P* < 0.05 is considered significant [14].

The sample size was calculated while assuming the incidence of difficult laryngoscopy to be 4% [15]. Based on the preliminary experiment, the sensitivity of the MMT and ULBT were 0.9231 and 0.2308, respectively. We determined that 450 patients would be required to demonstrate a difference between two predicting tools with a type 1 error (α) of 5% and power (1-β) of 90% (two-sided) using the PASS program.

## Results

**Fig. 1:**
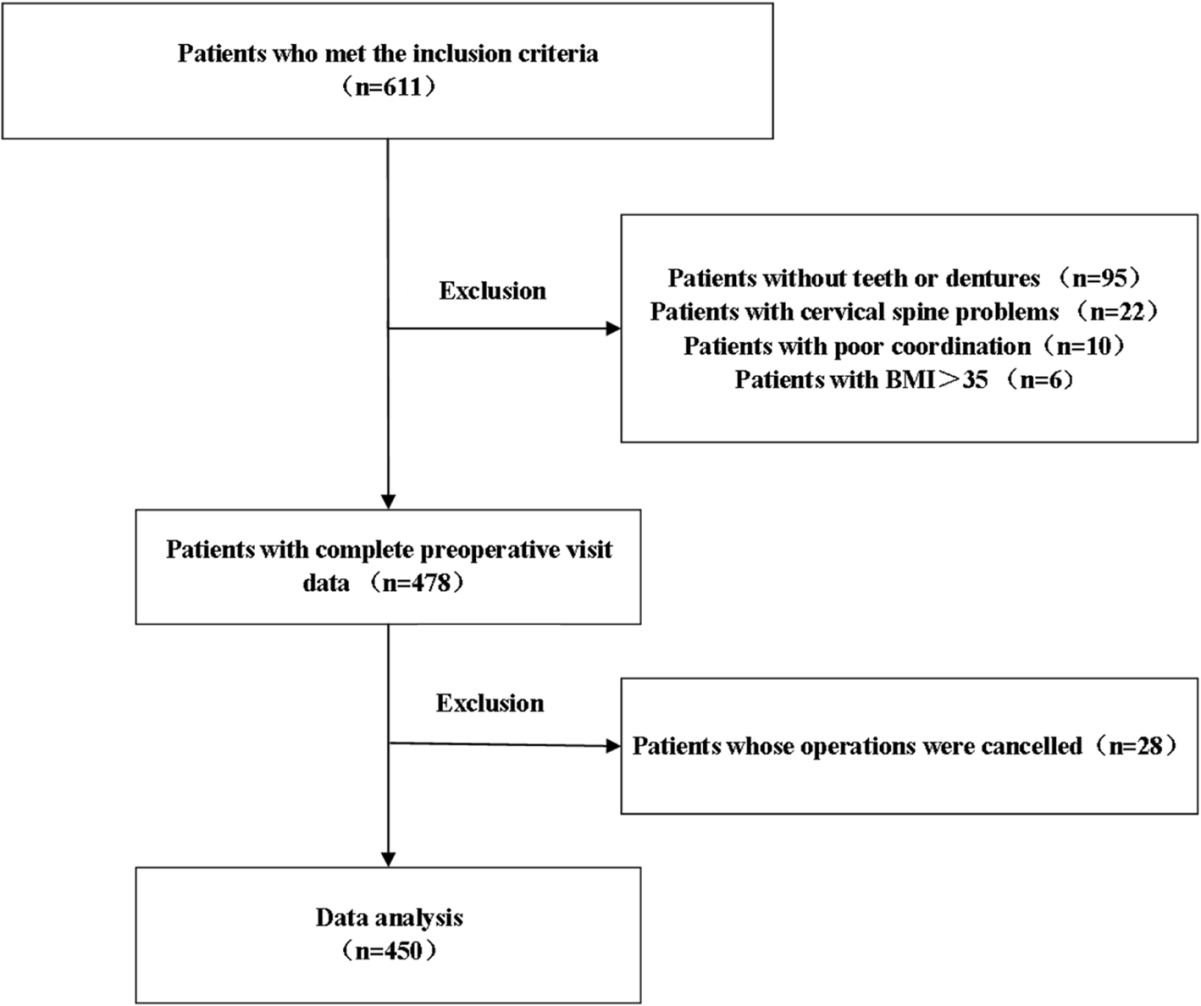
Flow chart of patient participation

A total of 611 patients with elective tracheal intubation and general anesthesia were enrolled in the study. One hundred thirty-three patients were excluded from preoperative visits due to lack of teeth or dentures, limited cervical mobility, poor coordination, and BMI greater than 35, and 28 patients were excluded due to cancelled operation for various reasons. Ultimately, data of 450 patients were analyzed (Fig. 1).

Two tests (ULBT and MMT) were performed on each patient. Of the 450 participants, 168 (37.3%) were classified as MMT Class I, 122 (27.1%) MMT Class II, 59 (13.1%) MMT Class III and 101 (22.4%) MMT Class IV. 250 (55.6%) participants were classified as ULBT Class I, 169 (37.6%) ULBT Class II and 31 (6.9%) ULBT Class III (Table 1).

**Table 1:** Frequency of MMT and ULBT grades and their contribution to difficult and easy intubation (in percentage)

Classes	Modified Mallampati Test (MMT)			Upper Lip Bite Test (ULBT)		
	Difficult intubation	Easy intubation	Total	Difficult intubation	Easy intubation	Total
	n (%)			n (%)		
Class I	12 (7.1%)	156 (92.9%)	168	35 (14.0%)	215 (86.0%)	250
Class II	14 (11.5%)	108 (88.5%)	122	26 (15.4%)	143 (84.6%)	169
Class III	13 (22.0%)	46 (78.0%)	59	8 (25.8%)	23 (74.2%)	31
Class IV	30 (29.7%)	71 (70.3%)	101			
Total (out of 450)	69 (15.3%)	381 (84.7%)	450	69 (15.3%)	381 (84.7%)	450

Cormack and Lehane’s (CL) class I & II are easy intubation and III & IV are difficult intubation

*ULBT* Upper lip bite test, *MMT* Modified Mallampati test

Of the 450 patients, 69 (15.3%) were classified as difficult cases of intubation: 59 (85.5%) CL III and 10 (14.5%) CL IV. Easy laryngoscopy was found in 381 (84.7%) patients; 155 (40.7%) CL I and 226 (59.3%) CL II. There were significant differences in mean age between difficult and easy laryngoscopy groups (*p* < 0.05), while weight, height, BMI, gender and ASA grade were not significant (*p* > 0.05) (Table 2).

**Table 2:** Demographic data

Variables	Intubation			***P***-value
	Overall (***n*** = 450)	Difficult (***n*** = 69)	Easy (***n*** = 381)	
Age (Years)	56.15 (10.91)	58.61 (10.55)	55.71 (10.93)	0.042*
Weight (kg)	68.14 (11.05)	70.109 (12.08)	67.781 (10.84)	0.108
Height (cm)	164.33 (7.78)	165.00 (7.67)	164.21 (7.80)	0.438
BMI (kg/m^2^)	25.16 (3.19)	25.68 (3.70)	25.07 (3.08)	0.196
Gender				0.073
Male	197 (43.78%)	37 (53.62%)	160 (41.99%)	
Female	253 (56.22%)	32 (46.38%)	221 (58.01%)	
ASA				1.000
I	22 (4.9%)	3 (4.3%)	19 (5.0%)	
II	428 (95.1%)	66 (95.7%)	362 (95.0%)	

Data are presented as mean (SD) and number (%)

CL Grade I & II are easy intubation and III & IV are difficult intubation

*significant (*p* < 0.05)

**Fig. 2:**
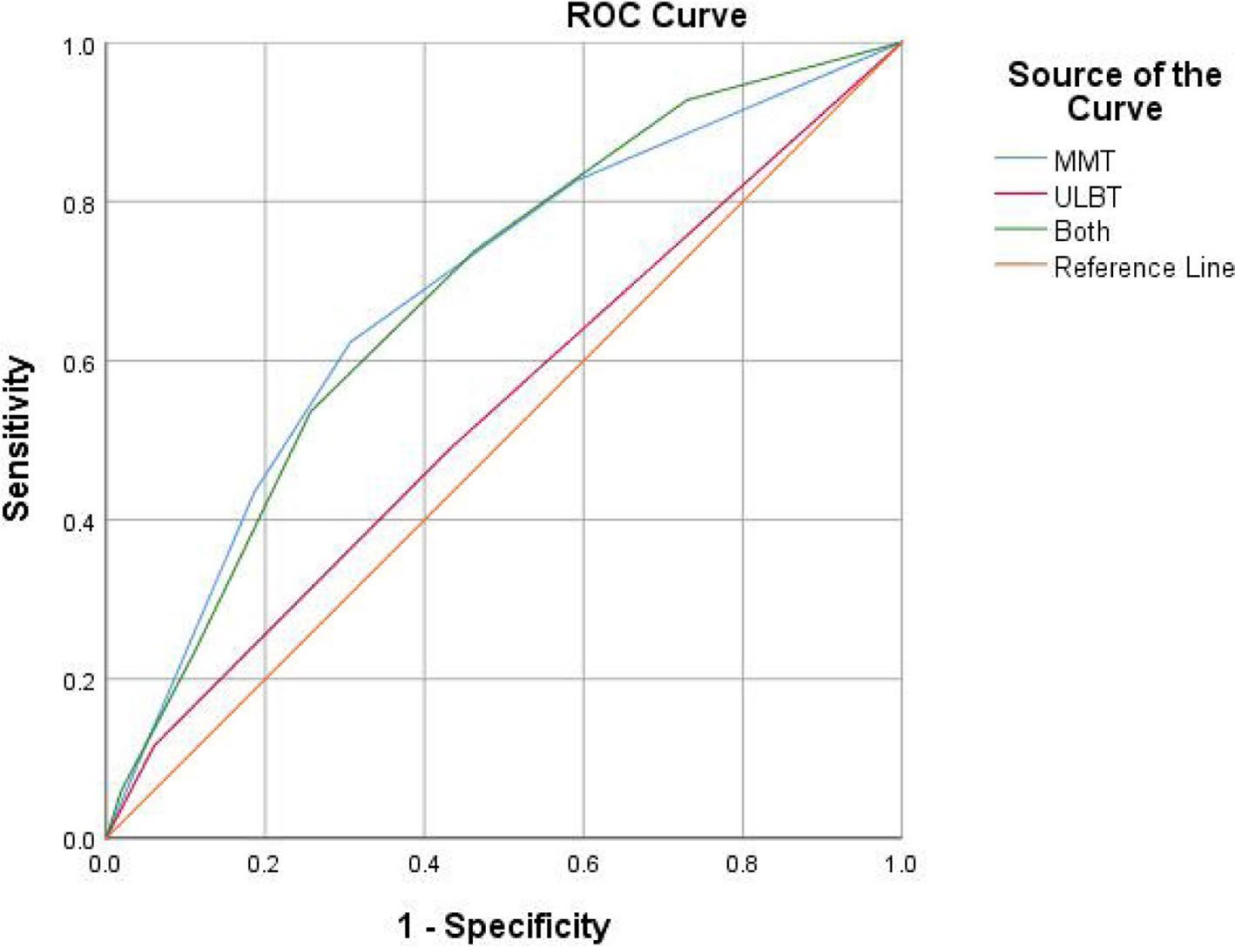
ROC curves for various tests. ULBT: Upper lip bite test; MMT: Modified Mallampati test; Both: The combination of ULBT and MMT

The accuracy, sensitivities, specificities, PPV, NPV, likelihood ratios, and area under ROC curve of the various tests for the prediction of difficult intubation are listed in Table 3 and Fig. 2.

**Table 3:** Predictive values for ULBT and MMT to predict difficult intubation according to CL classification

Predictive values	ULBT Estimates	MMT Estimates	ULBT + MMT Estimates
	[95%CI]		
Accuracy (%)	81.33	66.22	71.11
Sensitivity (%)	11.59	62.32	53.62
Specificity (%)	93.96	69.29	74.28
PPV (%)	25.81	26.88	27.41
NPV (%)	85.44	91.03	89.84
Likelihood ratio of a	1.92	2.03	2.08
Positive Test			
Likelihood ratio of a	0.94	0.54	0.62
Negative Test			
Kappa	0.072	0.205	0.039
Youden index	0.056	0.316	0.279
*P*-value	0.303	<0.001*	<0.001*
Area under ROC curve	0.539	0.684	0.684

*ULBT* Upper lip bite test, *MMT* Modified Mallampati test, *CI* Confidence interval, *PPV* Positive Predictive Value, *NPV* Negative Predictive Value

*significant (*p* < 0.05)

The *p*-value of paired Chi-square test (McNemar-Bowker Test) for the total score of MMT and 2-fold ULBT and CL grading was 0.128 (> 0.05) and Kappa value was 0.160 (< 0.4), indicating that there was no statistical difference between the prediction results of the two methods, but the consistency of the two methods was poor. Therefore, the total score of MMT and 2-fold ULBT couldn’t be used to directly predict CL grade under direct laryngoscopy. Similarly, the *p*-value of paired Chi-square test for the total score of ULBT and 2-fold MMT and CL grading was <0.001 (<0.05) and the Kappa value was 0.114 (<0.4), suggesting that the prediction results of the two ways were statistically different, but the consistency of the two ways was still not strong. The total score of ULBT and 2-fold MMT also did not directly predict CL grade under direct laryngoscopy (Table 4).

**Table 4:** Comparison of total scores of two different forms of ULBT and MMT with CL grading for predicting consistency of difficult intubation

		CL grading				McNemar-Bowker Test *P*-value	Kappa
		1	2	3, 4	Total		
ULBT×2 + MMT Scores	3—4	82	77	13	172	0.128	0.160
	5—7	55	120	38	213		
	8—10	18	29	18	65		
	Total	155	226	69	450		
MMT × 2 + ULBT Scores	3—5	99	113	20	232	<0.001*	0.114
	6—8	37	58	19	114		
	9—11	19	55	30	104		
	Total	155	226	69	450		

*ULBT* Upper lip bite test, *MMT* Modified mallampati test, *CL* Cormack and Lehane

*significant (*p* < 0.05)

In our study, the accuracy of ULBT (81.33%) was higher than that of MMT (66.22%), and the specificity of ULBT (93.96%) was higher than that of MMT (69.29%). In particular, in our trial, the sensitivity of the ULBT group (11.59%) was significantly lower than that of the MMT group (62.32%). The sensitivity, specificity, and accuracy of the combination of MMT and ULBT in the assessment of difficult intubation were between the two alone.

Among the 69 patients with difficult laryngoscopic view, the percentage of patients with MMT class > II was 62.3%(43 of 69), whereas only 8 patients (11.6%) had ULBT grade III.

## Discussion

Endotracheal intubation is an important means to maintain airway patency. A lack of necessary preparation for difficult airway can have disastrous consequences. It is always the goal of anesthesiologists to accurately judge the difficulty of intubation before operation.

Because ULBT requires no position restriction, no special equipment, no extra light and no voice restriction, which is very applicable, and the subjects can complete the test within a few seconds, it has become a popular bedside test for predicting difficult airways.

In previous tests, the comprehensive assessment of ULBT was a better predictor of difficult airways than MMT [5]. But current research results indicate that the clinical effect of ULBT is not superior to MMT, because sensitivity should be paid more attention to in judging and predicting the value of difficult intubation factors, so as not to miss patients with actual difficult airway and cause serious consequences.

In this study, the predicted results of the two tests, MMT and ULBT, were not as described in the study by Kahn et al. [5, 13, 16]. However, the most significant difference was that the sensitivity of the ULBT test in our trial was much lower. This ratio was only 11.6%, compared with 76.5% in the original experiment5, which was similar to some other studies [17–19]. This means that the ULBT test will fail to identify some patients with difficult airways (a large number of patients present with false negatives).

We concluded that one of the factors contributing to the low sensitivity of ULBT is the low incidence of ULBT class III in subjects. According to a summary of several literatures, this feature can be explained by skeletal variation and soft tissue redundancy in Far East Asians [17].

ULBT evaluates the range and degree of freedom of mandibular motion as well as the structure of teeth. In addition, ULBT is classified by the upper lip mucosa as the boundary. Therefore, any differences in these tissues will affect the results of ULBT. In the field of orthodontic and maxillofacial surgery, several studies comparing soft tissues of different ethnic groups have been published [20–24]. The anthropological literature described that craniofacial and dental alignment varies from race to race and confirmed that there are significant racial differences in mandibular and maxillary morphological measurements [25–27]. Thus ULBT may be a useful predictor in some populations, but its utility for Asians may be limited [17].

According to literature reports, the upper lip of Chinese people is longer and sharper than that of caucasians [23]. A morphometric analysis on European-American and Asian subjects was performed by Chang and colleagues [24]. Far East Asian (Chinese, Korean, Japanese, and Taiwanese) men have significantly shorter skulls and smaller anterior skull base angles. Due to the relative retraction of the naso-maxillary complex and the relative anteriorness of the mandible, Chinese people tend to have a shorter middle face, a protruding mandible and an anteriorly moved temporomandibular joint (TMJ) [17, 24]. Thus, the scarcity of grade III ULBT in Asians can be explained as a result of excessive soft lip tissues and an anterior TMJ.

The value of MMT in predicting difficult intubation has been controversial. In an extensive systematic evaluation of 34,513 patients in 42 studies, Lee et al. found MMT ranged in accuracy from poor to good [28]. In this study, we found the specificity of MMT to be 69.3% which was almost near to the study conducted by Khan et al. (66.8%) [5] and Eberhart et al. (61%) [18]. The sensitivity of MMT in this study is 62.3%, which is Lower than of *Jamuna* et al. (80%) [10]. Although the modified test largely solves the mouth opening and tongue base size problems associated with oropharynx, patient cooperation is critical and the test should be demonstrated well by observers. The anesthesiologist’s experience with intubation may also lead to changes in results.

In our trial, neither test could reproduce the high area under ROC curve and NPV of Khan et al. [5], but the two indicators of MMT were both higher than those of ULBT, indicating that MMT has higher diagnostic accuracy than ULBT.

The total scores of the two tests combined in different ways were less consistent with CL classification in predicting difficult intubation, which may be because the sensitivity of the two tests was not high, so the total scores were not a good predictor.

Both tests had high PPV, meaning that both predicted easy intubation very well, while NPV that predicted the incidence of difficult intubation were low. This means that other tests are needed to better predict difficult airways.

The main advantage of our study is that both tests were evaluated by the same investigator, and CL grading was also evaluated by experienced anesthesiologists, thus reducing the error of interobserver variation to a large extent.This study innovatively proposed to predict difficult airways using the total scores of the two tests, although the results showed that this simple and intuitive method was not feasible. The limitation of our study is that we were not able to test patients who were uncooperative or had problems with their teeth or cervical spine, so the results are not applicable to everyone. Larger sample size and more diverse population are needed to validate the value of ULBT and MMT in predicting difficult intubation under direct laryngoscopy.

## Conclusion

Although MMT and ULBT are easy to perform, they do not have high sensitivity, and misprediction results can lead to difficult intubation situations that are more dangerous. Both tests had high NPV and were better predictors of easy intubation rather than difficult intubation.The combination of ULBT and MMT in fractional form is also not a good predictor of intubation difficulties under direct laryngoscopy. Therefore, we should be more active in the search for more ideal tests and be prepared for the unexpected during anesthesia.

## Supplementary Information

**Additional file 1.**

## Data Availability

All data generated or analysed during this study are included in this published article [and its supplementary information files].

## Abbreviations

ULBT: Upper Lip Bite Test
MMT: Modified Mallampati Test
CL: Cormack and Lehane’s
PPV: Positive predictive value
NPV: Negative predictive value
LR: Likelihood ratio
ASA: American Society of Anesthesiologists
BMI: Body mass index
CI: Confidence interval
TMJ: Temporomandibular joint
